# Multidisciplinary Approach to Patients With Metastatic Spinal Cord Compression: A Diagnostic Therapeutic Algorithm to Improve the Neurological Outcome

**DOI:** 10.3389/fonc.2022.902928

**Published:** 2022-06-07

**Authors:** Rossella Rispoli, Chiara Reverberi, Giada Targato, Serena D’Agostini, Gianpiero Fasola, Marco Trovò, Mario Calci, Renato Fanin, Barbara Cappelletto

**Affiliations:** ^1^ SOC Chirurgia Vertebro-Midollare, Azienda Sanitaria Universitaria Friuli Centrale, Presidio Ospedaliero Universitario “Santa Maria della Misericordia” di Udine, Udine, Italy; ^2^ SOC Radioterapia, Azienda Sanitaria Universitaria Friuli Centrale, Presidio Ospedaliero Universitario “Santa Maria della Misericordia” di Udine, Udine, Italy; ^3^ SOC Oncologia, Azienda Sanitaria Universitaria Friuli Centrale, Presidio Ospedaliero Universitario “Santa Maria della Misericordia” di Udine, Udine, Italy; ^4^ SOC Neuroradiologia, Azienda Sanitaria Universitaria Friuli Centrale, Presidio Ospedaliero Universitario “Santa Maria della Misericordia” di Udine, Udine, Italy; ^5^ SOC Pronto Soccorso e Medicina d’Urgenza, Azienda Sanitaria Universitaria Friuli Centrale, Presidio Ospedaliero Universitario “Santa Maria della Misericordia” di Udine, Udine, Italy; ^6^ Clinica di Ematologia, Azienda Sanitaria Universitaria Friuli Centrale, Presidio Ospedaliero Universitario “Santa Maria della Misericordia” di Udine, Udine, Italy

**Keywords:** spinal metastasis, spinal cord compression, pathological spine fractures, diagnostic-therapeutic algorithm, neurological deficits

## Abstract

**Introduction:**

The morbidity associated with metastatic spinal disease is significant because of spinal cord and/or nerve root compression. The purpose of this paper is to define a diagnostic-therapeutic path for patients with vertebral metastases and from this path to build an algorithm to reduce the devastating consequences of spinal cord compression.

**Materials and Methods:**

The algorithm is born from the experience of a primary care center. A spine surgeon, an emergency room (ER) physician, a neuroradiologist, a radiation oncologist, and an oncologist form the multidisciplinary team. The ER physician or the oncologist intercept the patient with symptoms and signs of a metastatic spinal cord compression. Once the suspicion is confirmed, the following steps of the flow-chart must be triggered. The spine surgeon takes charge of the patient and, on the base of the anamnestic data and neurological examination, defines the appropriate timing for magnetic resonance imaging (MRI) in collaboration with the neuroradiologist. From the MRI outcome, the spine surgeon and the radiation oncologist consult each other to define further therapeutic alternatives. If indicated, surgical treatment should precede radiation therapy. The oncologist gets involved after surgery for systemic therapy.

**Results:**

In 2021, the Spine and Spinal Cord Surgery department evaluated 257 patients with vertebral metastasis. Fifty-three patients presented with actual or incipient spinal cord compression. Among these, 27 were admitted due to rapid progression of symptoms, neurological deficits and/or spine instability signs. The level was thoracic in 21 cases, lumbar in 4 cases, cervical in 1 case, sacral in 1 case. Fifteen were operated on, 10 of these programmed and 5 in emergency.

**Discussion:**

Patients with a history of malignancy can present to the ER or to the oncology department with symptoms that must be correctly framed in the context of a metastatic involvement. Even when there is no previous cancer history, the patient’s pain characteristics and clinical signs must be interpreted to yield the correct diagnosis of vertebral metastasis with incipient or current spinal cord compression. The awareness of the alert symptoms and the application of an integrated paradigm consent to frame the patients with spinal cord compression, obtaining the benefits of a homogeneous step-by-step diagnostic and therapeutic path. Early surgical or radiation therapy treatment gives the best hope for preventing the worsening, or even improving, the deficits.

**Conclusions:**

Metastatic spinal cord compression can cause neurological deficits compromising quality of life. Treatment strategies should be planned comprehensively. A multidisciplinary approach and the application of the proposed algorithm is of paramount importance to optimize the outcomes of these patients.

## Introduction

About 60% of secondary tumor localizations involves the spinal column (1). This is commonly believed to result from the large vascular supply and lymphatic drainage of vertebral bones (2). The progresses of chemo and radiation therapy treatments improved the survival of oncological patients and led to an increase of the number of patients with vertebral metastases (3, 4). Currently, spinal metastases are identified in approximately 20% of all oncological patients (5) and, among them, symptomatic spinal cord compression occurs in 25-50% (6–9). Cancers of the lung, breast, and prostate metastasize more frequently to the spine with a percentage that exceeds 60%; in about 7% of cases the primary tumor remains unknown (10).

Spinal cord compression occurs in 80% of patients with a known history of cancer and in the remaining 20% ​of cases, is the first manifestation of the tumor. These synchronous presentations are seen most frequently in lung cancer, but also in hematological malignances, like multiple myeloma and non-Hodgkin lymphomas, and require histological confirmation to plan the best therapeutic strategy (2, 9). Spine metastases with related neurological impairment are more often localized in the thoracic tract (11). The morbidity associated with metastatic spinal disease is significant. Subsequent mechanical instability and/or spinal cord or roots compression lead to paralysis, sensorial deficits and sphincter dysfunctions that impact on the quality of life and increase mortality.

Treatment of spinal metastases requires a multidisciplinary approach that integrates the knowledge of a team of specialists for prompt diagnosis of patients with spinal metastases and cord compression and optimal support after diagnosis. The purpose of this paper is to build an algorithm with the aim of reducing and preventing the irreversible neurological deficits and the devastating consequences of spinal cord compression.

## Materials and Methods

The institution where the algorithm was built is a primary care center. A team of specialists, emergency room (ER) physician, spine surgeon, neuroradiologist, radiation oncologist, and oncologist, got together and agreed on the crucial points and steps to follow.

The ER physician or the oncologist has the assignment to recognize the symptoms and signs of a metastatic spinal cord compression and has to trigger the next steps of the flow-chart. The alert symptoms are neck or back nocturnal pain, axial mechanical pain (induced or worsened by movements and under pressure relieved by lying down), sudden onset of axial pain, radicular pain radiating to arms or legs associated or not with numbness, tingling, dysesthesia, walking or balance difficulties or arms/hands weakness for impairment of one or more muscles, bladder or bowel control disorders, urinary retention (**Table 1**). These clinical manifestations induce the team’s physicians to follow the next step of the algorithm. If the symptoms are consistent with spinal cord compression, The spine surgeon takes charge of the patient, defines the neurological deficits and the appropriate timing for magnetic resonance imaging (MRI) in collaboration with the neuroradiologist. From the MRI outcome, the spine surgeon and the radiation oncologist consult each other to define further therapeutic alternatives. If indicated, surgical treatment should precede radiation therapy. The oncologist gets involved after surgery for systemic therapy. The proposed algorithm is illustrated in **Table 2**.

**Table 1:** Summary of the alert symptoms for metastatic roots or spinal cord compression (MSCC) and progression of metastatic spine disease.

- neck or back nocturnal pain - axial mechanical pain (induced or worsened by movements and under pressure relieved by lying down) - sudden onset of axial pain - radicular pain radiating to arms or legs associated or not with numbness, tingling, dysesthesia - walking or balance difficulties or arms/hands weakness for impairment of one or more muscles - bladder or bowel control disorders, urinary retention

**Table 2:** Diagnostic-therapeutic algorithm for patients with metastatic roots or spinal cord compression (MSCC).

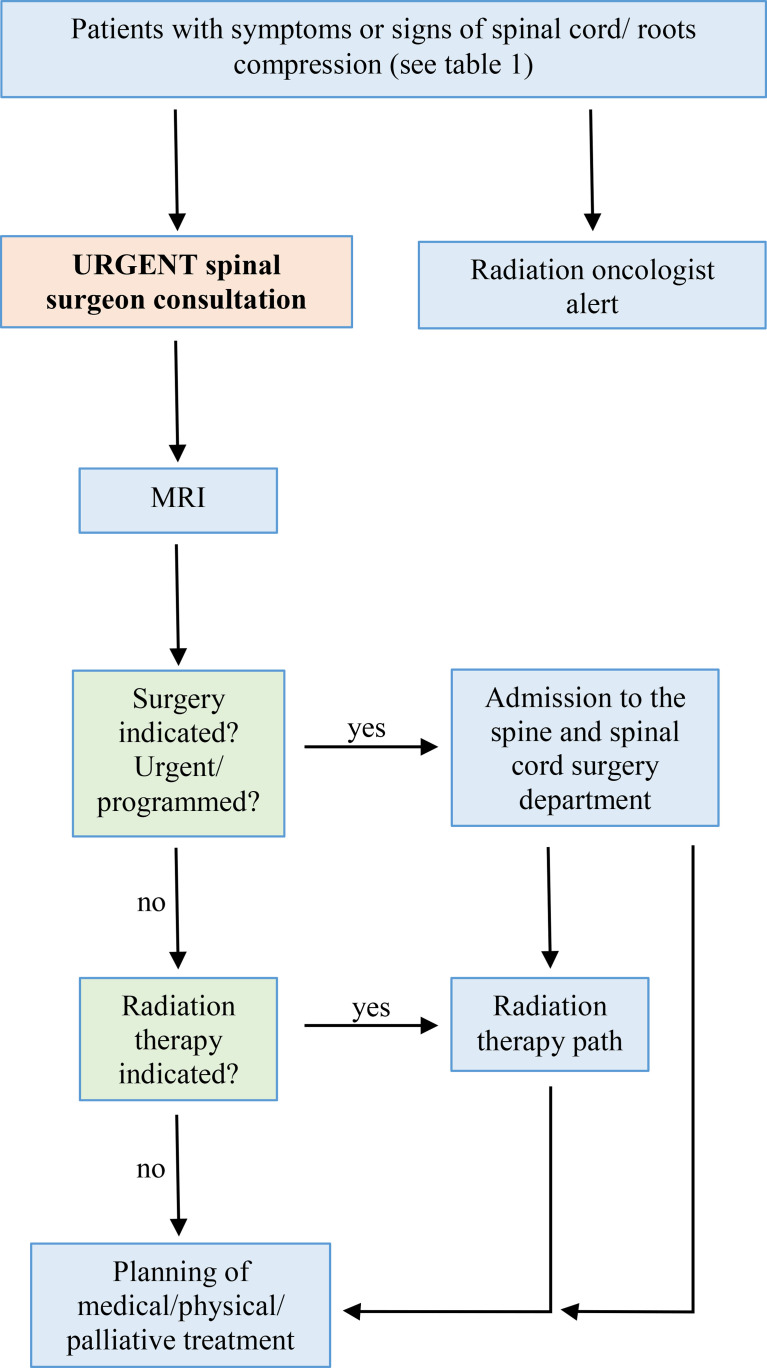	

MRI, magnetic resonance imaging.

The muscular strength is graded with the manual muscle testing (MMT) scale from 5 (normal) to 0 (no visible movement or palpable muscle contraction) (12). The Frankel grading system is used to summarize the functional grade of the patients (13). The neurological exam is completed with the sensory function and sphincter function evaluation.

## Results

In 2021, the Spine and Spinal Cord Surgery department evaluated 257 patients with vertebral metastasis. Fifty-three patients presented with actual or incipient spinal cord compression. Among these, 27 were admitted due to rapid progression of the symptoms, neurological deficits and/or spine instability signs; 14 were male and 13 female, mean age was 68.2 years. Breast (5 cases) and lung (4 cases) were the most frequent primitive cancer, followed by mesenchymal (3 cases), prostate (2 cases), kidney (2 cases), urothelial (2 cases), gastrointestinal (2 cases), hematologic (2 cases), neuroendocrine (1 case); in 4 cases the primitive was unknown. The level was thoracic in 21 cases, lumbar in 4 cases, cervical in 1 case, sacral in 1 case. Frankel grade at admission was A in 3 patients, B in 6 patients, C in 7 patients, D in 8 patients, and E in 3 patients.

Fifteen were operated on, 10 of these programmed and 5 in emergency.

The stratification and the characteristics of the patients are summarized in **Table 3** and in **Table 4**.

**Table 3:** Stratification of patients with vertebral metastases who were evaluated at the Spine and Spinal Cord Surgery department in the year 2021.

Number of patients evaluated with vertebral metastasis	Patients with actual or incipient spinal cord compression	Patients admitted with spine metastasis and neurological compression	Patients operated on for spine metastasis (total)	Patients operated on in emergency (<72h)
257	53	27	15	5

**Table 4:** Characteristics of the patients with spine metastasis and neurological compression admitted (n = 27) to the Spine and Spinal Cord Surgery department in the year 2021.

Gender distribution	Mean age	Primitive cancer of the patients admitted to the hospital	Level of the compression	Frankel grade at admission
14 males 13 females	68.2 years	5 breast, 4 lung, 3 mesenchymal, 2 prostate, 2 kidney, 2 urothelial, 2 gastrointestinal, 2 hematologic, 1 neuroendocrine 4 unknown	21 thoracic 4 lumbar 1 cervical 1 sacral	3 A 6 B 7 C 8 D 3 E

## Discussion

Metastases to the spine may be asymptomatic. Alternatively, patients with unknown metastatic disease could have nonspecific symptoms, including back pain. Due to the extraordinarily high frequency of back pain in middle age from a variety of root causes (14), the metastatic origin of the pain may be underestimated.

In the literature there are numerous algorithms on the treatment of spinal metastases but there are no formal protocols on how to prevent spinal cord compression. Communication and sharing information, as a means to establishing a multidisciplinary approach for the management of spine metastases in hospitals, is crucial.

### Alert Symptoms: Pain

The definition of alert symptoms is fundamental and is the first tool to identify patients at risk or with spinal cord compression. From 80 to 95% of patients with spinal metastases report spine pain as their first symptom (15). Pain can occur in different forms: localized, mechanical and radicular. Localized pain is related to periosteal inflammation, mechanical pain is suggestive of impending or established spinal instability, radicular pain may develop from nerve root compression by the tumoral tissue or secondary to vertebral collapse (16, 17). Localized spine pain is usually constant throughout the day, exacerbating at night or early morning, typically with posture changes, coughing or sneezing and lying flat (18). Sudden axial pain evokes a pathological fracture. Furthermore, the cancer pain could radiate through radicular districts. Patients with spine metastases can refer midscapular pain, band-like pain across the chest or hip pain, depending on the cervical, thoracic or lumbar localization of the metastases (18). Patients with a known diagnosis of neoplasm must be studied as soon as possible with whole spine MRI, with the hope of uncovering the metastases before compression occurs. Likewise, patients in apparent good health who show recent back pain must be examined as soon as possible (19). The first four parameters of the alert symptoms deal with pain that must be promptly recognized and framed (**Table 1**).

### Alert Symptoms: Neurological Deficits

Cord and root compression is characterized by motor, sensory and sphincter disorders (17, 18). Weakness and awkwardness in the movement of the limbs are the first signs of motor disturbance; dysesthesia and paresthesia indicate an initial sensory disturbance. Neurological symptoms and signs sometimes develop late, and they commonly call for urgent surgical treatment in order to preserve or improve the residual neurological functions (20–22). The last two parameters of the alert symptoms deal with the neurological deficits that must be properly evaluated (**Table 1**).

### Diagnosis: MRI

Once framed correctly, on the base of the alert symptoms, the patient is evaluated by the spinal surgeon who decides the timing of performing the MRI which is superior to all other imaging modalities in its uncovering of spinal metastases. MRI provides essential information about spinal cord and nerve root compression.

The study protocol requires the MRI exam of the whole spine. The MRI determines the extent of the disease both in terms of a single vertebra and in terms of the number of vertebrae involved. The exam is able to show the compression or infiltration of the spinal cord and nerve roots. It is essential to carry out sagittal T1 and T2 weighted MRI sequences of the whole spine and axial T2-weighted sequences of the affected spinal levels. Spinal metastases are usually hypointense on T1 sequences; they can be hypo- or hyper-intense on T2 MRI sequences depending on their blastic or lytic characteristics, respectively. A fat suppression sequences such as T2-weighted short-tau inversion recovery (STIR) is useful to better define the metastatic lesions (23). Diffusion-weighted sequences can also be used to enhance the diagnostic accuracy in particular for the differential diagnosis with other alterations of the vertebral signal, often present and concomitant in the cancer patient (osteoporosis, bone marrow reconversion) (24). Contrast enhancement is not required to demonstrate spinal bone metastasis, but it can be useful if spinal cord localization or leptomeningeal metastatic infiltration is suspected (24, 25).

### Algorithms for Patient Management

Several guidelines for spine metastases recommend that clinicians pay great attention to the early signs of metastatic spinal cord compression and advise an early diagnosis through the execution of the MRI examination of the whole spine (26). Some studies have demonstrated that specific systems developed for earlier diagnosis and treatment can decrease treatment delays, which is in turn associated with improved neurological outcomes of patients. Some authors report that delayed treatment leads to a worse surgical and post-operative outcome (surgical timing, blood loss, length of stay and postoperative adverse events) with a negative influence on the patient’s quality of life (27, 28). Allan et al. proposed a system to detect early symptoms of spine metastases through a telephone interview with cancer patients performed in order to define the most appropriate timing for an MRI examination. This process reduced the timing of the diagnosis, improved outcomes and the appropriateness of the MRIs (29). Savage et al. reported that the formalization of a system for providing fast access to MRI derived from the collaboration between specialists can improve outcomes, agreeing with the National Institute for Health and Care Excellence (NICE) guidance (30). Nakata et al. established a multidisciplinary approach with the aim of providing an urgent MRI and referral to the spine surgeon in order to reduce or avoid neurological deficits caused by metastatic spinal cord compression (31). In our algorithm, if the symptoms are consistent with spine metastases, the spine surgeon defines the appropriate timing for MRI in collaboration with the neuroradiologist. The awareness of the alert symptoms and the application of an integrated paradigm create a rapid, essential portrait of patients with spinal cord compression. Compared to other systems, ours benefits from both a homogeneous step-by-step diagnostic (early whole spine MRI) and therapeutic (early surgery or radiation therapy) path.

### Guidelines Treatment

The spine is a complex system from an anatomical, biomechanical, neurological point of view; for this reason, the treatment of spinal metastases is more challenging than that of other bones. There are no homogeneously applied guidelines for spinal metastases but there is the unanimous opinion that this disease must be treated simultaneously by several specialists (32).

Before planning a treatment, the patient’s performance status, the cancer type, the systemic burden of disease and availability of effective systemic treatment options must be considered. The possible benefits to be accrued from any treatment should be carefully weighed against the morbidity and risks involved. The Spine Oncology Consortium (SOC) has divided the treatment options for spinal metastasis into three categories – radiotherapy, surgery and neurointerventional procedures – that can be applied simultaneously, consequentially and/or individually (22). Frameworks for decision making in regard to spine metastases management such as the neurological, oncological, mechanical and systemic (NOMS) and the location, mechanical instability, neurology, oncology and patient’s factors (LMNOP) have been developed (18, 33). LMNOP is the most used algorithm to determine a therapeutic strategy (34). The Spine Oncology Study Group developed the Spinal Instability Neoplastic Score (SINS) to determine the degree of instability associated with a spinal metastasis. With this system, specialists and non-specialists can directly judge the spine instability (35, 36). In general, invasive locoregional treatments may be preferentially considered in patients with better prognosis. In patients with poor performance status (≤40%) and with less than two months of life expectancy, the multidisciplinary team should preferentially consider best supportive care (22). Since there is no consensus to specify what life expectancy justifies a surgical intervention, the NOMS working group reported that the surgical option should not be excluded *a priori* in patients with low life expectancy but should be the object of a multidisciplinary discussion. This discussion should address the likelihood of the patient recovering from surgery and thereby continuing systemic anticancer treatment (21).

In addition to the tumor burden, the histology and biology of a tumor is a strong prognostic element and is also important in guiding the choice of treatment to be pursued. According to literature, some tumors (Hodgkin and non-Hodgkin lymphomas, germ-cell neoplasm, myelomas, neuroblastoma, prostate and breast cancer) present high chemo and/or radiosensitivity. For these cancer types, a medical and/or a radiation treatment might be preferred over surgery (21, 22). On the contrary, other tumors (non-small cell lung cancer, colon carcinoma and carcinoma of unknown primary origin) showed radio-resistance and, in some series, short survival outcomes after spine surgery and thus the benefit from extensive intervention is less marked (37).

### Radiation Therapy

Symptomatic patients with documented metastatic spinal cord compression not suitable for surgery, must be urgently referred to the radiation oncologist in order to be treated with radiotherapy (38). The optimal timing of treatment delivery from the onset of symptoms is within 24-72 hours. According to the speed of onset, duration, severity of neurological symptoms, patient’s performance status and prognosis, radiotherapy can be offered as definitive treatment. It could be fractionated, generally 20 Gray (Gy) in 5 fractions and 30 Gy in 10 fractions, or a single fraction of 8 Gy. No differences in clinical outcome, defined as motor function improvement, were described. Nevertheless, the long-term outcomes showed better local controlled disease in patients who received a longer radiotherapy course (39). A preliminary report from the single-fraction radiotherapy compared to multifraction radiotherapy (SCORAD) randomized phase III trial recommend the use of single fraction over 5 fractions in patients with short-term prognosis (median survival 3 months) (40). Several studies demonstrated that urgent radiotherapy delivered as 8 Gy single fraction is generally the best therapeutic regimen for symptoms palliation, even when the patient is completely paralyzed. Moreover, further radiotherapy can be considered for patients who reacted well to previous treatment. The NICE guidelines suggest fractionated radiotherapy should be considered for patients having good prognosis (38). Patients with complete neurologic deficit for more than 72 hours or poor prognosis are not candidates for urgent radiotherapy. Pre-operative radiotherapy is not a standard of care, whereas post-operative radiotherapy can be offered to patients having a good surgical outcome. Fractionated radiotherapy can be offered in the adjuvant setting, once the surgical scar is completely recovered. The most common radiotherapy schedule is 30 Gy in 10 fractions.

Surgery and radiotherapy are the cornerstones of metastatic spinal cord compression treatment. Whether to prefer one to the other approach is a complex decision, requiring a multidisciplinary approach. The decision-making process takes into account patients’ prognosis, performance status and comorbidity, grade of neurological functions and spine instability. Patchell et al. (41) reported that a larger percentage of patients treated with surgery and adjuvant radiotherapy had better outcome and remained ambulatory (84% vs 57%, p = 0.001) compared to the patients treated with radiotherapy alone.

### Surgery

Surgery aims to decompress of the neural structures, to locally remove the tumor (separation surgery), and to afford the stability of the spine (42). Some authors recommend surgery only if the patient has a life expectancy longer than 3 months. Although some minimally invasive procedures to decompress and stabilize the spine can be offered to the patients with severe root pain or axial pain due to instability, independently from other variables (34, 42). According to many authors, minimally invasive surgery should be considered the first-choice treatment in patients with metastatic spinal compression. It has many advantages, such as shortening the surgical time, reducing the trauma of soft tissues and blood losses, consenting early mobilization, shortening the length of stay in hospital and good pain control. All these factors favor a greater speed in starting the adjuvant treatment, providing the patient with a greater therapeutic possibility (43–45). Laminectomy without stabilization is no longer used because it can create iatrogenic instability (46). However, in selected cases of tumor involving only the posterior elements or epidural tumor without bone involvement, laminectomy is a reasonable surgical option. Separation surgery is a technique that has the objective to create a safe distance between the spinal cord/roots and the tumor that will be then treated with radiation therapy (47, 48). Spine stereotactic radiosurgery (SRS) is increasingly considered as a first-choice treatment when possible so that *en bloc* removal is less used in recent years. More innovative materials, like poly-ether-ether-ketone (PEEK) and carbon-fiber, are used in order to reconstruct the vertebral body and create fewer artifacts in radiological images to favor radiotherapy techniques (49, 50). Even more recently, CT guided three-dimensional printing of plastic polymers or titanium constructs, is being developed to create customized supports for patients (51).. Robot-assisted guidance and spine navigation provide greater precision and definition in tumor removal and placement of pedicle screws (52).

### Medical, Physical, Palliative Treatment

The oncologist cares for the patient after the surgical or/and radiation therapy treatment and defines the subsequent follow-up and the appropriate systemic anticancer treatment, tailored on patient and cancer characteristics.

Lastly, rehabilitation, bracing and muscular strengthening can improve the patient’s quality of life. Analgesia steroids, drugs for neuropathic pain and bisphosphonates can be used if necessary (52, 53).

## Conclusions

Spine metastases cause serious morbidities, such as pathological fractures, spinal cord/root compression, and neurological deficits. Our hospital, a primary care center, has developed an algorithm that defines the parameters useful for avoiding metastatic spinal cord compression and improving the patients’ outcome.

The expectation for 2022 is to verify the effectiveness of the methodology introduced in the integrated care pathway. We plan to identify and check the following key performance indicators (KPI):

1) Time elapsed between first consultation (emergency room) and the MRI
2) Time from MRI to start of treatment (surgery or radiation therapy)
3) Grade of neurological deficits (Frankel scale) at the time of their recognition and at follow-up.

The future objective is to statistically analyze and compare the parameters listed above among the two groups, i.e. patients of the year 2021, without the application of the algorithm, and patients of the year 2022, after application of the algorithm.

## Data Availability Statement

The raw data supporting the conclusions of this article will be made available by the authors, without undue reservation.

## Ethics Statement

Ethical review and approval was not required for the study on human participants in accordance with the local legislation and institutional requirements. The patients/participants provided their written informed consent to participate in this study.

## Author Contributions

Authors are responsible for the entire content of each article. Co-authorship should be based on the following four criteria: (1) substantial contributions to the conception or design of the work; or the acquisition, analysis, or interpretation of data for the work; (2) drafting of the work or revising it critically for important intellectual content; (3) final approval of the version to be published; and (4) agreement to be accountable for all aspects of the work and ensuring that questions related to the accuracy or integrity of any part of the work are appropriately investigated and resolved.

Each author must affirm that they participated in and contributed sufficiently to the work to take public responsibility for the following: (1) conception and design, (2) data acquisition, (3) analysis of data, (4) drafting of the manuscript, (5) critical revision, (6) obtaining funding, (7) administrative support, or (8) supervision.

## Conflict of Interest

The authors declare that the research was conducted in the absence of any commercial or financial relationships that could be construed as a potential conflict of interest.

## Publisher’s Note

All claims expressed in this article are solely those of the authors and do not necessarily represent those of their affiliated organizations, or those of the publisher, the editors and the reviewers. Any product that may be evaluated in this article, or claim that may be made by its manufacturer, is not guaranteed or endorsed by the publisher.
